# Impact of oxygen tension according to embryo stage of development: a prospective randomized study

**DOI:** 10.1038/s41598-021-01488-9

**Published:** 2021-11-16

**Authors:** C. Herbemont, J. Labrosse, B. Bennani-Smires, I. Cedrin-Durnerin, M. Peigne, N. Sermondade, S. Sarandi, A. Vivot, E. Vicaut, Z. Talib, M. Grynberg, C. Sifer

**Affiliations:** 1 Department of Cytogenetic and Reproductive Biology, Hôpital Jean Verdier, Avenue du 14 Juillet, 93140 Bondy, France; 2 Department of Reproductive Medicine & Fertility Preservation, Hôpital Jean Verdier, 93140 Bondy, France; 3 Department of Cytogenetic and Reproductive Biology, Hôpital Tenon, 4 rue de la Chine, 75020 Paris, France; 4 Clinical Epidemiology Unit, Hôtel Dieu, 1 Parvis Notre-Dame, 75004 Paris, France; 5 Unité de Recherche Clinique, Hôtel Lariboisière-Fernand Widal, 200 rue du Faubourg Saint Denis, 75010 Paris, France

**Keywords:** Medical research, Outcomes research

## Abstract

Human embryo culture under 2–8% O2 is recommended by ESHRE revised guidelines for good practices in IVF labs. Nevertheless, notably due to the higher costs of embryo culture under hypoxia, some laboratories perform embryo culture under atmospheric O2 tension (around 20%). Furthermore, recent meta-analyses concluded with low evidence to a superiority of hypoxia on IVF/ICSI outcomes. Interestingly, a study on mice embryos suggested that oxidative stress (OS) might only have an adverse impact on embryos at cleavage stage. Hence, we aimed to demonstrate for the first time in human embryos that OS has a negative impact only at cleavage stage and that sequential culture conditions (5% O2 from Day 0 to Day 2/3, then «conventional» conditions at 20% O2 until blastocyst stage) might be a valuable option for human embryo culture. 773 IVF/ICSI cycles were included in this randomized clinical trial from January 2016 to April 2018. At Day 0 (D0), patients were randomized using a 1:2 allocation ratio between group A (20% O2; n = 265) and group B (5% O2; n = 508). Extended culture (EC) was performed when ≥ 5 Day 2-good-quality-embryos were available (n = 88 in group A (20% O2)). In subgroup B, 195 EC cycles were randomized again at Day 2 (using 1:1 ratio) into groups B’ (5% O2 until Day 6 (n = 101)) or C (switch to 20% O2 from Day 2 to Day 6 (n = 94). Fertilization rate, cleavage-stage quality Day 2-top-quality-embryo (D2-TQE), blastocyst quality (Day 5-top-quality-blastocyst (D5-TQB) and implantation rate (IR) were compared between groups A and B (= cleavage-stage analysis), or A(20% O2), B’(5% O2) and C(5%-to-20% O2). Overall, characteristics were similar between groups A and B. Significantly higher rates of early-cleaved embryos, top-quality and good-quality embryos on Day 2 were obtained in group B compared to group A (*P* < 0.05). This association between oxygen tension and embryo quality at D2 was confirmed using an adjusted model (*P* < 0.05). Regarding blastocyst quality, culture under 20% O_2_ from Day 0 to Day 6 (group A) resulted in significantly lower Day 5-TQB number and rates (*P* < 0.05) compared to both groups B’ and C. Furthermore, blastocyst quality was statistically equivalent between groups B’ and C (*P* = 0.45). At Day 6, TQB numbers and rates were also significantly higher in groups B’ and C compared to group A (*P* < 0.05). These results were confirmed analyzing adjusted mean differences for number of Day 5 and Day 6 top quality embryos obtained in group A when compared to those respectively in groups B’ and C (*P* < 0.05). No difference in clinical outcomes following blastocyst transfers was observed. These results would encourage to systematically culture embryos under hypoxia at least during early development stages, since OS might be detrimental exclusively before embryonic genome activation.

## Introduction

In mammals, the oxygen (O2) tension within the intratubal and intrauterine environments is low (between 2 and 8%)^1,2^. Hence, human embryo development is supposed to be optimal under similar conditions. However, embryo culture under hypoxia (5%) is not yet standardized, as embryos are cultured under atmospheric O2 tension (around 20%) in up to 25% of laboratories performing Assisted Reproductive Technology (ART) procedures^3^. Several randomized studies on human embryos have reported the superiority of hypoxia (5%) in terms of embryo quality and blastulation rates^4–7^. Since oxidative stress (OS) might have a negative impact on embryo development^8^, these results might be explained by the fact that hypoxia enables a more physiological environment that induces less OS. Other studies have also suggested that OS damages might be irreversible before compaction^9,10^. Interestingly, a study investigating the impact of oxygen tension on embryo development in mice found that culture either exclusively at 5% O2 or sequential culture at 5% from Day 0 to Day 2, then at 20% from Day 2 to Day 4 did not significantly affect blastulation, suggesting that oxidative stress might only impact cleavage stage, and that switching culture to atmospheric conditions from Day 2/3 might not influence embryo development thereafter^11^.

Hence, these investigations suggest that embryo culture using tri-gas incubators (5% O2, 6% CO2 and 89% N2) should be preferred. Besides, the latest guidelines published by the European Society of Human Reproduction and Embryology (ESHRE) recommended low O2 tension for embryo culture^12^. However, tri-gas culture systems require important N2 consumption (around 7L/hour in benchtop incubators) and more complicated logistics (e.g. N2 levels monitoring), making it more expensive than standard incubation conditions. Yet, Wale and Gardner’s findings^11^ imply that sequential culture conditions (tri-gas from Day 0 to Day 2/3, then « conventional » conditions at 20% O2 until blastocyst stage) could be a valuable option, reducing the costs and possibly without any detrimental impact on embryo development. However, the impact of sequential culture conditions has never been investigated in human embryos so far. The present study has two main objectives: (i) to confirm that low oxygen tension improves embryo quality and (ii) to demonstrate for the first time in human embryos that OS has a negative impact only at cleavage stage, as assumed by Wale and Gardner^11^.

## Results

### Cleavage-stage analysis

Characteristics of patients randomized at Day 0 are summarized in Table 1. Overall, demographic, infertility and stimulation data were similar between groups A (20% O2) and B (5% O2). Only the total dose of FSH administered was significantly higher in group B (2 582 *vs.* 2 319 IU in groups B and A, respectively; *P* < 0.01). Considering biological parameters, the statistical analysis outlined significantly higher rates of early-cleaved embryos, of top-quality and good-quality embryos on Day 2 in group B compared to group A (early-cleaved embryos: 38.8% *vs.* 26.6%; top-quality embryos: 41.0% *vs.* 36.7%; good-quality embryos: 58.0% *vs.* 53.6%; *P* < 0.05, respectively). This association between oxygen tension and embryo quality at Day 2 was confirmed in a multivariate model studying mean differences. This last analysis reported an absolute number of Day 2-top-quality embryos and D2-good-quality embryos in favor of group B, respectively increased by + 0.60 and + 0.64.

**Table 1:** Demographic and clinical characteristics of patients randomized at Day 0.

	Group A 20% O2 (n = 265)	Group B 5% O2 (n = 508)
**Female age (y)**	32.8 (4.1)	32.9 (4.3)
**Male age (y)**	36.3 (6.4)	36.9 (6.8)
**Female BMI (kg/m**^**2**^**)**	24.5 (4.4)	24.7 (4.2)
**Male BMI (kg/m**^**2**^**)**	26.4 (4.0)	26.1 (4.3)
**Smoking (female), n (%)**		
Yes	54 (22.0)	80 (17.0)
**Antral follicle count (n)**		
AMH (ng/mL)	4.2 (3.1)	4.2 (5.0)
Day 3 FSH (IU/L)	6.7 (2.1)	6.6 (2.2)
**Ovarian function, n (%)**		
Normal	214 (86.0)	389 (83.0)
Infertility duration (y)	4.4 (2.2)	4.7 (2.7)
**Cause of infertility, n (%)**		
Female	138 (48.0)	252 (50.0)
Male	94 (35.0)	219 (43.0)
Idiopathic	74 (28.0)	105 (21.0)
**Stimulation protocol (%)**		
Antagonist	238 (90.0)	433 (85.0)
Total FSH dose administered (IU)	2319 (1102)	2582 (1228)*
Estradiol day hCG (pg/mL)	2604 (1399)	2457 (1241)
Progesterone day hCG (pg/mL)	1.0 (0.6)	1.0 (0.8)
Endometrium thickness (mm)	10.3 (2.4)	10.7 (2.6)

*y* years, *n* number, *BMI* body mass index.

Data are presented as mean (SD) or n (%).

*P < 0.01.

No significant difference was observed between the two groups in terms of clinical outcomes, both in univariate and multivariate models.

### Blastocyst analysis

The impact of oxygen tension on later embryo development was evaluated in a 3-group analysis including all extended culture cycles performed in group A (20% O2) and group B, the latter being randomized a second time in groups B’ (still 5% O2) or C (20% O2 starting from Day 3). Patient and cycle characteristics are described in Table 2, showing no other significant difference between the three groups apart from the total dose of FSH administered.

**Table 2:** Demographic and clinical characteristics of patients randomized at Day 2 (included in« blastocyst analysis »).

	Group A 20% O2 (n = 88)	Group B’ 5% O2 (n = 101)	Group C 5–20% O2 (n = 94)
**Female age (y)**	32.8 (3.8)	32.2 (4.6)	33.3 (4.5)
**Male age (y)**	35.7 (6.0)	36.2 (6.7)	37.6 (7.3)
**Female BMI (kg/m**^**2**^**)**	24.9 (4.4)	25.2 (4.3)	25.2 (4.6)
**Male BMI (kg/m**^**2**^**)**	26.4 (3.7)	26.5 (4.6)	25.4 (4.1)
**Smoking (female), n (%)**			
Yes	12 (15.0)	16 (17.0)	16 (18)
**Antral follicle count (n)**			
AMH (ng/mL)	4.8 (3.3)	4.8 (4.6)	5.3 (6.9)
Day 3 FSH (IU/L)	6.1 (2.0)	6.2 (1.6)	6.0 (1.8)
**Ovarian function, n (%)**			
Normal	65 (76.0)	74 (79.0)	63 (73)
Infertility duration (y)	4.6 (2.3)	4.9 (2.8)	4.4 (2.5)
**Cause of infertility, n (%)**			
Female	57 (65.0)	49 (49.0)	57 (61)
Male	24 (27.0)	39 (39.0)	33 (35)
Idiopathic	16 (18.0)	22 (22.0)	14 (15)
**Stimulation protocol (%)**			
Antagonist	81 (92.0)	90 (89.0)	86 (91)
**Total FSH dose administered (IU)**	2005 (823)	2348 (1060)	2351 (1285)*
**Estradiol day hCG (pg/mL)**	2870 (1747)	2530 (1184)	2760 (1412)
**Progesterone day hCG (pg/mL)**	0.9 (0.7)	0.9 (0.5)	0.8 (0.6)
**Endometrium thickness (mm)**	10.3 (2.4)	10.6 (2.3)	11.0 (2.4)

Data are presented as mean (SD) or n (%).

*Global *P*-value < 0.05.

Blastulation rates were similar between groups A, B’ and C (67.9% *vs.* 69.8% *vs.* 72.4%, respectively) (Table 5). However, the number and rate of top-quality blastocysts at Day 5 were significantly higher in groups B’ (n = 2.6, 24.4% under 5% O2) and C (n = 2.7, 22.2% under 5–20% O2) when compared to group A (n = 1.9, 17.5% under 20% O2, *P* < 0.05), respectively. Interestingly, these parameters were not statistically different whether embryo culture was continued under 5% O2 (group B’) or 20% O2 (group C) after Day 2 (Table 5). When focusing on embryo quality at Day 6, the number and rate of top-quality blastocysts were again significantly higher in group B’ (3.0, 33.7%) and C (3.0, 31.4%) compared to group A (n = 2.1, 26.4%, *P* < 0.05) (Table 3). These results were confirmed analyzing adjusted mean differences for numbers of Day 5 (-0.94, -0.82) and Day 6-top-quality embryos (-1.12, -0.89) obtained in group A when compared to those respectively in groups B’ and C (*P* < 0.05) (Table 4).

**Table 3:** Comparison of biological outcomes between groups A, B’ and C (20% *vs.* 5–5% *vs.* 5–20% O2, respectively).

	Group A 20% O2 (n = 88)	Group B’ 5% O2 (n = 101)	Group C 5–20% O2 (n = 94)
Number of embryos/cycle	12.3 (5.4)	11.2 (4.7)	12.5 (6.0)
Number of blastocysts/cycle	8.0 (3.4)	7.7 (3.8)	8.8 (4.2)
% Blastulation	67.9 (19.2)	69.8 (21.2)	72.4 (18.4)
**Day 5**			
Number of top-quality blastocysts % of top-quality blastocysts/embryo Number of good-quality blastocysts % good-quality blastocysts /embryo	1.9 (1.6) 17.5 (14.7) 2.9 (1.8) 27.0 (17.6)	2.6 (2.0) 24.4 (18.6) 3.3 (2.1) 30.7 (19.2)	2.7 (2.2)* 22.2 (16.6)* 3.6 (2.4) 29.9 (17.4)
**Day 6**			
Number of top-quality blastocysts % of top-quality blastocysts/embryo Number of good-quality blastocysts % good-quality blastocysts /embryo	2.1 (1.6) 26.4 (16.5) 3.2 (3.8) 29.6 (18.3)	3.0 (2.1) 33.7 (20.7) 3.8 (2.3) 34.1 (18.9)	3.0 (2.4)* 31.4 (19.9)* 3.8 (2.5) 31.8 (18.3)
Number of embryos transferred	1.4 (0.5)	1.4 (0.5)	1.5 (0.5)
Usable embryo rate, %	50.1 (20.3)	52.8 (21.5)	51.9 (19.7)

Data are presented as mean (SD).

* Global *P*-value < 0.05.

**Table 4:** Adjusted mean differences and 95% CI from linear model with adjustment for multiple comparisons between groups A, B’ and C.

Group	A *vs.* B’	A *vs.* C	B ’ *vs.* C
**Day 5**			
Number of top-quality blastocysts	− 0.94 (− 1.62, − 0.26)*	− 0.82 (− 1.52, − 0.13)*	0.12 (− 0.55, 0.79)
Number of good-quality blastocysts	− 0.67 (− 1.40, 0.05)	− 0.62 (− 1.36, 0.12)	0.05 (− 0.66, 0.77)
**Day 6**			
Number of top-quality blastocysts	− 1.12 (− 1.86, − 0.38)*	− 0.89 (− 1.65, − 0.14)*	0.23 (− 0.50, 0.95)
Number of good-quality blastocysts	− 0.79 (− 1.58, − 0.01)	− 0.59 (− 1.39, 0.21)	0.20 (− 0.57, 0.98)

*P < 0.05.

Finally, clinical outcomes following blastocyst transfers were comparable whatever the oxygen tension applied during culture, as described respectively in Table 5 for univariate and Table 6 for multivariate models.

**Table 5:** Comparison of clinical outcomes following blastocyst transfer between groups A, B’ and C.

	Group A 20% O2 (n = 88)	Group B’ 5–5% O2 (n = 101)	Group C 5–20% O2 (n = 94)
Number of transfers	56	80	61
Implantation rate	42.9 (45.2)	36.9 (46.2)	41.8 (46.7)
Biochemical pregnancy	62.5 (35)	47.5 (38)	50.8 (31)
Clinical pregnancy	51.8 (29)	41.3 (33)	47.5 (29)
Miscarriage	22.8 (8)	21.0 (8)	16.1 (5)

Data are presented as mean (SD) or % (n).

**Table 6:** Adjusted Odds-ratio and 95% CI from logistic model with adjustment for multiple comparisons of clinical outcomes following blastocyst transfer between groups A, B’ and C.

Group	A *vs.* B’	A *vs.* C	B’ *vs.* C
Biochemical pregnancy	1.76 (0.70–4.43)	1.28 (0.48–3.46)	0.73 (0.30–1.78)
Clinical pregnancy	1.70 (0.67–4.31)	1.12 (0.41–3.03)	0.66 (0.26–1.64)
Miscarriage	0.50 (0.10–2.51)	1.11 (0.18–6.76)	2.23 (0.40–12.52)

## Discussion

To our best knowledge, this original study is the first to show that atmospheric oxygen tension has no impact on human blastocyst quality, as long as the first 2 days of embryo development occur under hypoxia.

The analysis performed on the first days of embryo development confirmed the harmful impact of atmospheric oxygen on embryo morphology at cleavage-stage, which had been previously described^7,13–15^. Indeed, both top-quality embryo and good-quality embryo rates at Day 2 were significantly higher when embryos were cultured under hypoxia, as well as the rate of early cleavage. Interestingly, this latter point had never been described before, either not evaluated at all or not reaching statistical significance^10^. Several prospective studies^16–18^ had already defined embryo early cleavage as a good-prognosis marker of embryo morphology and clinical outcomes (improving good-quality embryo rates, implantation rates and clinical pregnancy rates). Therefore, the significant enhancement of this parameter highlighted under 5% O2 emphasizes the interest of culturing embryos in hypoxia to optimize ART outcomes.

When considering the “blastocyst analysis”, blastulation rates were comparable in the three groups (20% O2 *vs.* 5% O2% *vs.* 5–20% O2). Our results are in line with those previously published^4,7^. However, the impact of oxygen concentration on blastulation rates remains controversial. Indeed, most studies reporting an impact of oxygen tension focused on supernumerary embryos but not on embryos left in extended culture from the outset^15,19^, which makes their conclusions poorly reliable.

By contrast, our study revealed a significant improvement in blastocyst quality at Day 5 and Day 6 when embryo culture was performed under hypoxia exclusively (group B’) compared to 20% O2 exclusively (group A), confirming the conclusions published by Kovacic et al.^13^. Interestingly, higher top-quality blastocyst rates were also obtained when culture was performed under hypoxia from Day 0 to Day 2 followed by atmospheric conditions from Day 2 onwards (group C). Moreover, either exclusive hypoxia or sequential culture yielded similar results in terms of top-quality blastocyst rates, suggesting that oxidative stress might be less harmful on embryo development after compaction. This observation is consistent with those published by Wale and Gardner on mice embryos^11^. Conversely, studies following a similar but opposite design (sequential culture under 20% O2 from Day 0 to Day 2/3 followed by 5% O2 until Day 5/6) showed that the defects caused by oxidative stress on early embryo stages could not be reversed by lowering oxygen tension from Day 2 or Day 3^9,20^.

Differences in the metabolism of pre- and post-compaction embryos according to oxygen tension have already been described in mice^21^. Briefly, a physiological oxygen tension was associated to a lower pyruvate uptake and amino-acids turnover in pre-compaction embryos, which seems consistent with the “quiet embryo hypothesis” described at this stage^22^. Hence, the higher pyruvate consumption under atmospheric conditions could reflect greater antioxidant needs since pyruvate displays antioxidant properties. Conversely, after compaction, a higher glucose and amino-acid uptake was evidenced under hypoxia, which is also consistent with the increase in biosynthetic activity related to blastulation. However, the exact mechanisms through which post-compaction embryos better tolerate oxidative stress remain to be elucidated. One hypothesis could be a favorable antioxidant/oxidant balance due to hypoxia during the first days of development, leaving a sufficient stock for the post-compaction embryo to combat oxidative stress induced by atmospheric oxygen tension. Further studies at the metabolic level would be required to precise embryo response to oxidative stress under such conditions.

If hypoxia does not appear as a tool to produce more embryos, it could be used to produce more top-quality embryos, notably blastocysts. It is admitted that morphological parameters of blastocysts (expansion, inner cell mass and trophectoderm morphology) are directly correlated to euploidy rates^23,24^ as well as pregnancy outcomes^25,26^. However, clinical outcomes described in the present study are similar whatever the oxygen tension applied during embryo culture. This is in contradiction with other randomized controlled trials that concluded to an improvement in implantation rates and clinical pregnancy rates when transferring Day 3 embryos cultured in hypoxia^7,15^. The similar implantation rates obtained in our study can possibly be explained by the fact that blastocysts to be transferred were the top-ranked of the entire embryo cohort. The follow-up of cumulative clinical outcomes including subsequent frozen embryo transfers should technically demonstrate an improvement in success rates in case of embryo culture under hypoxia, as recently reported^27^.

Our study displays several methodological key strengths. It included one of the largest sample sizes focusing on embryo development until blastocyst stage and a homogeneous selected population due to the randomized design. Indeed, the few differences highlighted, namely the total gonadotropin dose administered during ovarian stimulation, can be considered negligible since a freeze-all policy was applied as soon as a risk of ovarian hyperstimulation syndrome was detected. Thus, no impact on implantation potential due to suboptimal endometrial receptivity could be suspected in our analysis. Another methodological key point of this study is the use of similar benchtop incubators in each group. Thus, culture conditions were strictly comparable except for gas environment. Indeed, some of the studies evaluating the impact of oxygen on embryo development either describe the use of different kind of incubators (e.g. large-box incubators in atmospheric conditions and benchtop incubators in hypoxia)^15,20,28^, or do not specify the type of incubators used^4^. Large-box incubators are less stable than benchtop incubators, causing additional stress to embryos because of temperature and pH variations, and possibly affecting the conclusions of such studies.

Physiologically, a drop in oxygen concentration from the Fallopian tube (5–7%) to the uterine cavity (2%) has been described^2^. As a consequence, the use of a sequential oxygen concentration (5%-to-20% O2) in our study contrasts with recent publications evaluating the interest of applying ultra-low oxygen tension during human embryo culture (2% O2). However, methodological flaws, such as the use of tripronucleate embryos^29^ or D3 frozen-thawed embryos after culture under atmospheric conditions at cleavage-stage^30^, lessen the relevance of their conclusions. More recently, a prospective study on sibling oocytes failed to find any difference, neither in blastulation rates nor in blastocyst quality, between a continuous culture under 5% O2 and a sequential culture (5% from D0 to D3, switched to 2% O2 from D3 to D5/6)^31^. Nevertheless, the publication of meta-analyses demonstrating with high evidence the superiority of hypoxia over atmospheric oxygen tension on both biological and clinical outcomes seems necessary before concluding to a potential benefit of ultra-low O2 tension.

In conclusion, hypoxia during human embryo culture improves embryo quality and development ability. However, late pre-implantation embryo development seems to be less vulnerable to oxidative stress. If confirmed, these results would encourage to systematically culture embryos under hypoxia at least during early development stages, since oxidative stress might be detrimental exclusively before embryonic genome activation. The clinical impact of such procedures in our study needs to be further analyzed considering the cumulative pregnancy rate.

## Methods

### Trial design

A prospective randomized interventional study was conducted in the assisted reproductive technique (ART) unit of Jean Verdier University Hospital (Bondy, France), after being approved by the Institutional Revision Board (Comité de Protection des Personnes Ile De France X: EmbryOx- N° ID-RCB: 2015-A02019-40) and registered on Clinicaltrial.gov (trial registration date: 23/05/2019; NCT03964805). All methods were performed in accordance with relevant guidelines and regulation and the study was conducted according to institutional and ethical rules regarding research on patients.

### Participants

A total of 773 couples undergoing IVF/ICSI were included between January 2016 and April 2018. All patients met the following inclusion criteria: (i) female age < 40 years on the day of oocyte retrieval; (ii) IVF/ICSI cycle using fresh or frozen ejaculated sperm from the male partner; (iii) at least 8 oocytes retrieved in total. Patients with one or more hydrosalpinges were not included in the study.

A first analysis was performed on Day 2 embryos (cleavage-stage analysis). Then, a second analysis was performed on blastocysts for embryos who had extended culture.

### Interventions

Each couple fulfilling these criteria was informed on Day 0 after oocyte retrieval and gave their written informed consent to be enrolled in the study. A double randomization between patients was performed.

On Day 0, two groups were defined (randomization 1:2, « cleavage-stage analysis »):

- Group A: embryo culture was performed at 20% O2 from Day 0.
- Group B: embryo culture was performed at 5% O2.

**Figure 1:**
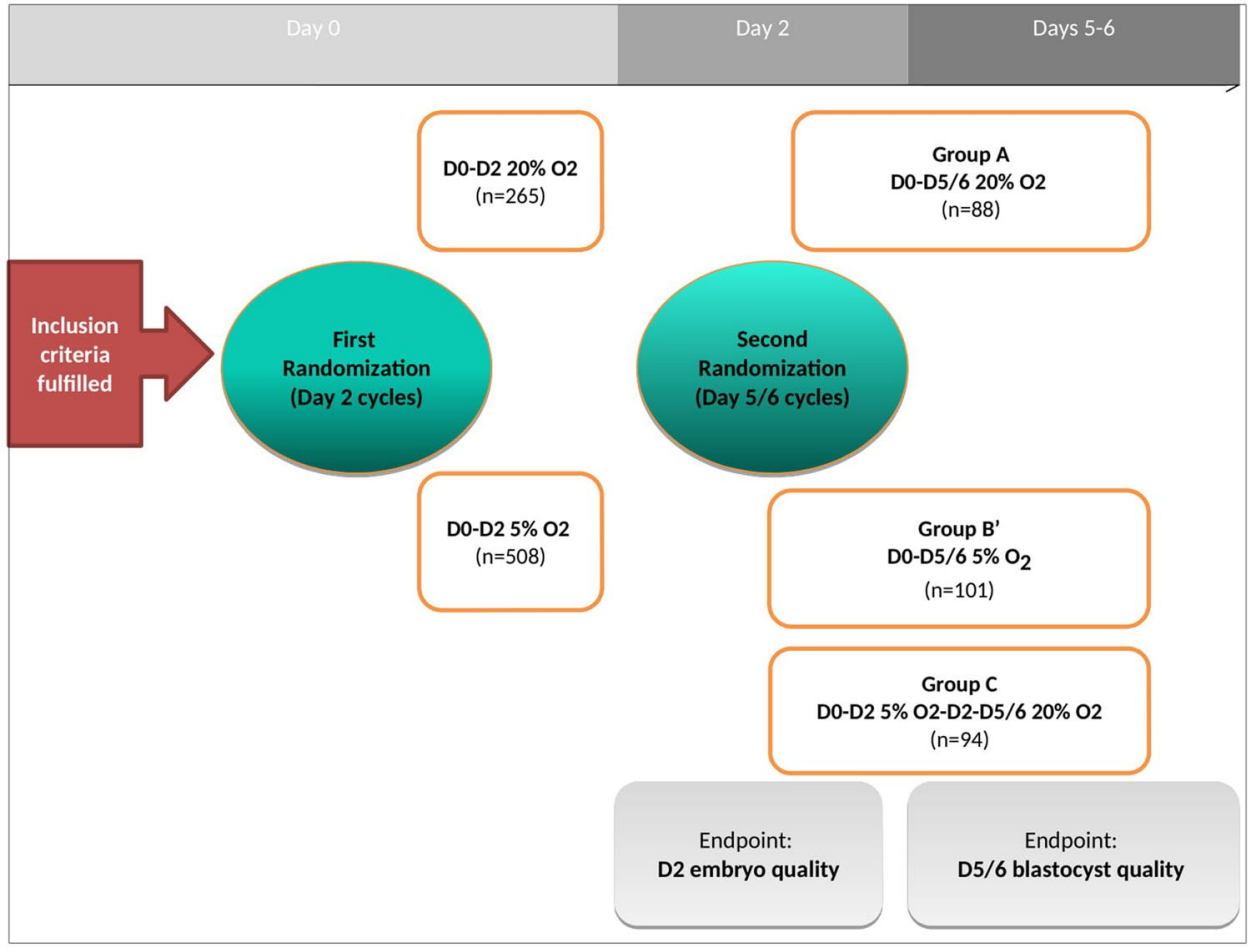
Flowchart.

On Day 2, extended culture was offered to good-prognosis patients (when at least 5 Day 2 good-quality embryos were available). For those in group B only (5% O2) who accepted culture to the blastocyst stage, a second randomization between patients was performed (randomization 1:1, “blastocyst analysis”), leading to (Fig. 1):

- Group B’: culture exclusively at 5% O2 from Day 0 to Day 6
- Group C: sequential culture, at 5% O2 from Day 0 to Day 2, then switched to 20% O2 from Day 2 to Day 6

Both were compared to extended cultures performed in group A (20% O2 from Day 0 to Day 6).

### IVF procedures

Briefly, ejaculated semen was prepared using a two-layer density technique. Controlled ovarian hyperstimulation was conducted with standard agonist or antagonist protocols. Administration of hCG was performed as soon as ≥ 3 preovulatory follicles (16–22 mm in diameter) were observed and E_2_ levels per preovulatory follicle were > 200 pg/mL. Oocyte retrieval occurred 36 h after hCG administration by transvaginal ultrasound-guided aspiration. Conventional in vitro fertilization (IVF) and intracytoplasmic sperm injection (ICSI) procedures were performed 3-6 h after oocyte pick-up. Embryos were cultured until D6 in an appropriate culture media equilibrated at 37 °C in a 6% CO_2_ atmosphere in a benchtop incubator (G-210, K-Systems, CooperSurgical, Denmark), either under atmospheric O2 (20% O2), or under hypoxia (5% O2, 89% N2). The medium had been equilibrated to oxygen tension during 4 h prior to adding embryos. Assessment of fertilization, defined by the presence of two pronuclei (2PN) and two polar bodies, was performed using an inverted microscope (× 200 magnification) 18 h after insemination (conventional IVF) or sperm injection (ICSI). Embryo quality (qualified as the number of blastomeres, degree of cytoplasmic fragmentation, regularity of the cells and presence/absence of multinucleated blastomeres) was evaluated at Day 2.

Embryos were either transferred at cleavage-stage (Day 2) or left in extended culture until Day 6 for good-prognosis cases. At Day 5/Day 6, blastocyst morphology was evaluated following Gardner and Schoolcraft’s classification^32^. Blastocysts were scored according to size: B1, early blastocyst with a blastocoel less than half the volume of the embryo; B2, with a blastocoel ≥ half the volume of the embryo; B3, full blastocyst with a blastocoel completely filling the embryo; B4, expanded blastocyst with a blastocoel volume larger than that of the blastocyst, and a thinning zona; B5, hatching blastocyst with a TE starting to herniate through the zona; and B6, fully hatched blastocyst. For blastocysts graded B3 to B6, the development of the inner cell mass (ICM) and TE was assessed. The ICM was graded as: A, tightly packed, many cells; B, loosely grouped, several cells; C, very few cells. The TE was graded as: A, many cells forming a tightly knit epithelium; B, few cells; C, very few cells forming a loose epithelium.

Fresh embryo/blastocyst transfers were performed under ultrasound guidance, at Day 2 or Day 5.

Luteal phase substitution was initiated using vaginal micronized progesterone (Progestan® Besins international, Montrouge, France) 200 mg twice a day from the evening after oocyte retrieval. This treatment was continued at the same dose until the pregnancy blood test, then until the 10-12th gestational week in case of pregnancy.

### Outcomes

The primary endpoint of this study was embryo quality, assessed at Day 2 and at Day 5 and Day 6 for patients included in the extended culture analysis. Day 2 top-quality embryos were defined as 4 regular blastomeres, < 20% cytoplasmic fragmentation, no multinucleations; Day 2 good-quality embryos as 3–5 cells, < 20% cytoplasmic fragmentation, no multinucleations; Day 5 and Day 6 top quality blastocysts were defined as ≥ B4AA/AB/BA according to Gardner and Schoolcraft^32^ and Day 5/Day 6 good-quality blastocysts were defined as ≥ B3BB.

Secondary endpoints were biological parameters including fertilization rate (percentage of oocytes fertilized per oocyte inseminated, assessed at Day 1), early cleavage rate (percentage of embryos at the 2-cell stage per oocyte fertilized, assessed 25 h after insemination), useable embryo rate (percentage of embryos transferred and/or frozen per embryo), and clinical outcomes including implantation rate (number of gestational sacs with fetal heart beat detected per embryo transferred), clinical pregnancy rate (percentage of pregnancies diagnosed by ultrasonographic visualization of at least one gestational sac with fetal heart beat per embryo transfer) and miscarriage rate (defined as the loss of a biochemical or clinical pregnancy before 20 completed weeks of gestational age).

### Sample size calculation

A difference of 5% has been reported by Ciray et al*.*^9^ between the rate of good-quality blastocysts (per D2 embryo) cultured under 5% O2 (good-quality blastocyst rate = 16%) and under 20% O2 (good-quality blastocyst rate = 11%). Therefore, considering an *alpha* risk = 0.05 and a power = 0.90, the sample size required was at least of 2 085 blastocysts in total, i.e. 695 per group. On average, in our ART center, each cycle with an extended culture (performed when at least 8 oocytes are retrieved) produces 8.4 blastocysts. Therefore, at least 83 cycles per group (i.e. 249 cycles in total) were required for the extended culture analysis. The percentage of cycles with an extended embryo culture procedure is of 33% in our current practice. Hence, a total of 755 cycles is required to assess the impact of O2 tension at the early cleavage stages of embryo culture.

### Randomization and allocation of patients to study groups

Couples were assigned to one of the study groups after eligibility was established and patient consent was obtained. Randomization was created using a computer-generated randomization list with 1:2 allocation for the first randomization, and 1:1 for the second randomization, using random block size of 10.

### Statistical analysis

Quantitative data were described as mean and standard deviation and compared by one-way ANOVA for the comparison between three groups. Qualitative data were described by n and percentage and compared by chi-square test or Fisher’s exact test when the validity of the test was not reached. All analyses were performed on R software, version 3.1. Outcomes were analyzed between groups by fitting a logistic model adjusted on female age, body mass index (BMI), number of previous IVF attempts, infertility duration, total dose of gonadotropins and number of metaphase 2 stage oocytes. *P*-values were adjusted for multiple comparisons using the Hochberg method. *P*-values < 0.05 were considered statistically significant.

## Data Availability

The data underlying this article will be shared on reasonable request to the corresponding author.
